# A Novel GMP Protocol to Produce High-Quality Treg Cells From the Pediatric Thymic Tissue to Be Employed as Cellular Therapy

**DOI:** 10.3389/fimmu.2022.893576

**Published:** 2022-05-16

**Authors:** Esther Bernaldo-de-Quirós, Beatriz Cózar, Rocío López-Esteban, Maribel Clemente, Juan Miguel Gil-Jaurena, Carlos Pardo, Ana Pita, Ramón Pérez-Caballero, Manuela Camino, Nuria Gil, María Eugenia Fernández-Santos, Susana Suarez, Marjorie Pion, Marta Martínez-Bonet, Rafael Correa-Rocha

**Affiliations:** ^1^ Laboratory of Immune-Regulation, Gregorio Marañón Health Research Institute (IISGM), Madrid, Spain; ^2^ Cell Culture Unit, Gregorio Marañón Health Research Institute (IISGM), Madrid, Spain; ^3^ Pediatric Cardiac Surgery Unit, Hospital Materno Infantil Gregorio Marañón, Madrid, Spain; ^4^ Pediatric Heart Transplant Unit, Hospital Materno Infantil Gregorio Marañón, Madrid, Spain; ^5^ Cell Production Unit, Gregorio Marañón Health Research Institute (IISGM), Madrid, Spain

**Keywords:** Treg, thymus, thyTreg, immunotherapy, GMP manufacturing, tolerance induction

## Abstract

Due to their suppressive capacity, the adoptive transfer of regulatory T cells (Treg) has acquired a growing interest in controlling exacerbated inflammatory responses. Limited Treg recovery and reduced quality remain the main obstacles in most current protocols where differentiated Treg are obtained from adult peripheral blood. An alternate Treg source is umbilical cord blood, a promising source of Treg cells due to the higher frequency of naïve Treg and lower frequency of memory T cells present in the fetus’ blood. However, the Treg number isolated from cord blood remains limiting. Human thymuses routinely discarded during pediatric cardiac surgeries to access the retrosternal operative field has been recently proposed as a novel source of Treg for cellular therapy. This strategy overcomes the main limitations of current Treg sources, allowing the obtention of very high numbers of undifferentiated Treg. We have developed a novel good manufacturing practice (GMP) protocol to obtain large Treg amounts, with very high purity and suppressive capacity, from the pediatric thymus (named hereafter thyTreg). The total amount of thyTreg obtained at the end of the procedure, after a short-term culture of 7 days, reach an average of 1,757 x10^6^ (range 50 x 10^6^ – 13,649 x 10^6^) cells from a single thymus. The thyTreg product obtained with our protocol shows very high viability (mean 93.25%; range 83.35% – 97.97%), very high purity (mean 92.89%; range 70.10% – 98.41% of CD25^+^FOXP3^+^ cells), stability under proinflammatory conditions and a very high suppressive capacity (inhibiting in more than 75% the proliferation of activated CD4^+^ and CD8^+^ T cells *in vitro* at a thyTreg:responder cells ratio of 1:1). Our thyTreg product has been approved by the Spanish Drug Agency (AEMPS) to be administered as cell therapy. We are recruiting patients in the first-in-human phase I/II clinical trial worldwide that evaluates the safety, feasibility, and efficacy of autologous thyTreg administration in children undergoing heart transplantation (NCT04924491). The high quality and amount of thyTreg and the differential features of the final product obtained with our protocol allow preparing hundreds of doses from a single thymus with improved therapeutic properties, which can be cryopreserved and could open the possibility of an “off-the-shelf” allogeneic use in another individual.

## Introduction

The immune system is the body’s defense mechanism against pathogens and other harmful agents. However, it is also responsible for transplant rejection or autoimmune diseases, in which an exacerbated response to the graft or one’s cells develops. Autoimmune diseases have a very high incidence affecting 4-8% of the population of developed countries. The graft-versus-host disease (GVHD), which is the leading cause of mortality and morbidity after hematopoietic cell transplantation, is also associated with excessive or undesired immune responses. Another scenario of disproportionate immune response is the cytokine release syndrome (CRS), a systemic inflammatory response characterized by a sharp increase of proinflammatory cytokines triggered by factors such as infections, drugs, chimeric antigen receptor T cell (CAR-T) therapy in oncologic patients, or GVHD (1). The ongoing COVID-19 pandemic has brought out CRS’s fatal consequences caused by SARS-CoV-2 infection, being one of the leading causes of death in severe patients (2).

In most cases, the standard treatment to prevent these immune responses is the use of immunosuppressive drugs, but they still do not provide a definitive solution and produce side effects that are decisive in the patient’s clinical course. Because immunosuppressants have a pleiotropic action, the entire immune system is suppressed, affecting the ability to defend the host against infections and the development of tumors or promoting autoimmune disorders. Besides, this strategy will always have the immune system’s degradation and generalized chronic damage as a counterpart. Therefore, a widespread feeling among the scientific community is that only re-educating the immune system to promote immune tolerance will reduce the harmful immune responses without damaging the immune system’s functional integrity. In this sense, cellular therapies based on the infusion of cells capable of inducing tolerance is generating great enthusiasm in the clinical practice, being regulatory T cells (Treg), a subtype of CD4^+^ T cells with suppressive function, the most studied and promising alternative (3). The immunoregulatory capacity of Treg cells is not due to one particular suppression mechanism, but rather it is the set of several coordinated mechanisms capable of promoting immune regulation. Indeed, Treg can suppress the effector function of a wide range of cells, including CD4^+^ and CD8^+^ T cells, B cells, dendritic cells, macrophages, granulocytes, natural killer cells, and osteoclasts (4). The mechanisms they use to suppress the different immune cells can be considered direct when the Treg themselves are the ones that provoke a response directly on the target cell, or indirect, in which another cell or molecule is affected, leading to the suppression of the target cell (5). The crucial role of Treg in preventing the hyperactivation of the immune system has been confirmed in transplanted adults (6) and children (7–9), GVHD (10), Crohn’s disease (11) and other autoimmune disorders (12–14). Therefore, a therapeutic strategy based on Treg cells could offer excellent results in the prevention or treatment of these diseases to significantly increase their number in circulation and enhance the intrinsic mechanisms of tolerance in these patients.

Except for the employment of Treg derived from cord blood in GVHD, all clinical trials and therapeutic approaches administering Treg cells follow the same design: Treg are purified from peripheral blood from the patient or donor, expanded *ex vivo* and reinfused as an autologous or allogeneic therapy respectively (15). The safety and potential efficacy of Treg therapy in humans is reflected in clinical trials already conducted, that show that peripheral or cord blood Treg infusion reduces or prevents various immune disorders such as GVHD or type 1 diabetes in the short term (16–19). The greatest risk of GVHD occurs during the first three months, and immune suppression by Treg therapy during this short critical period has been shown to be sufficient to provide long-term tolerance. However, in the case of solid organ transplants, the risk of rejection persists throughout the patient’s life, which makes necessary a protective effect of Treg that would last over time to ensure the prevention of graft rejection. There are currently numerous clinical trials in progress in phase I or phase I/II that use Treg cell therapy to prevent rejection of the transplanted organ in adult patients, most of them in the context of kidney and liver transplantation (20, 21). However, to date, the efficacy results are not entirely conclusive, mainly due to the low or short therapeutic effect of the infused Treg (22, 23). This could be due to the low number of Treg that can be purified from peripheral blood and the relative quality of infused Treg inherent to their higher state of cellular differentiation, which could be worsened after extensive *ex vivo* expansion (24). Although umbilical cord blood is a promising source of Treg cells, compared to adult peripheral blood due to the relatively high frequency of naïve Treg cells and the scarcity of memory T cells (25), the number of Treg that can be recovered of a single cord blood unit is very low. This fact implies that these naïve Treg have to undergo many rounds of *ex vivo* expansion to generate enough cells for a clinical dose which could also have an impact on their quality (24, 26).

Therefore, the search for an alternative source of Treg with a predominantly naïve state that allows obtaining enough quantity of cells is crucial to overcome the limitations encountered in peripheral blood and umbilical cord blood. In this sense, the thymus, a primary lymphoid organ responsible for the maturation and differentiation of T and Treg cells, which is located above the heart and discarded to gain access to the heart during pediatric cardiac surgeries, could be employed as a new source of highly undifferentiated Treg (27, 28).

## Materials and Methods

### Thymic Tissue Obtention

Human thymuses used for this research were excised and discarded in pediatric cardiac surgeries at the Pediatric Cardiac Surgery Unit of Gregorio Marañón Hospital (HGUGM). Thymic tissue was collected in sterile containers with TexMACS GMP medium (Miltenyi Biotec) supplemented with 1% antifungal antibiotic (Penicillin-streptomycin-amphotericin B; Sigma-Aldrich) and kept at 4°C until processing. The study was conducted after the HGUGM ethics committee’s approval and according to the principles expressed in the Declaration of Helsinki. Informed written consent from the legal guardians was obtained before the patient’s enrolment.

### ThyTreg Production in the Research Laboratory

**Figure 1:**
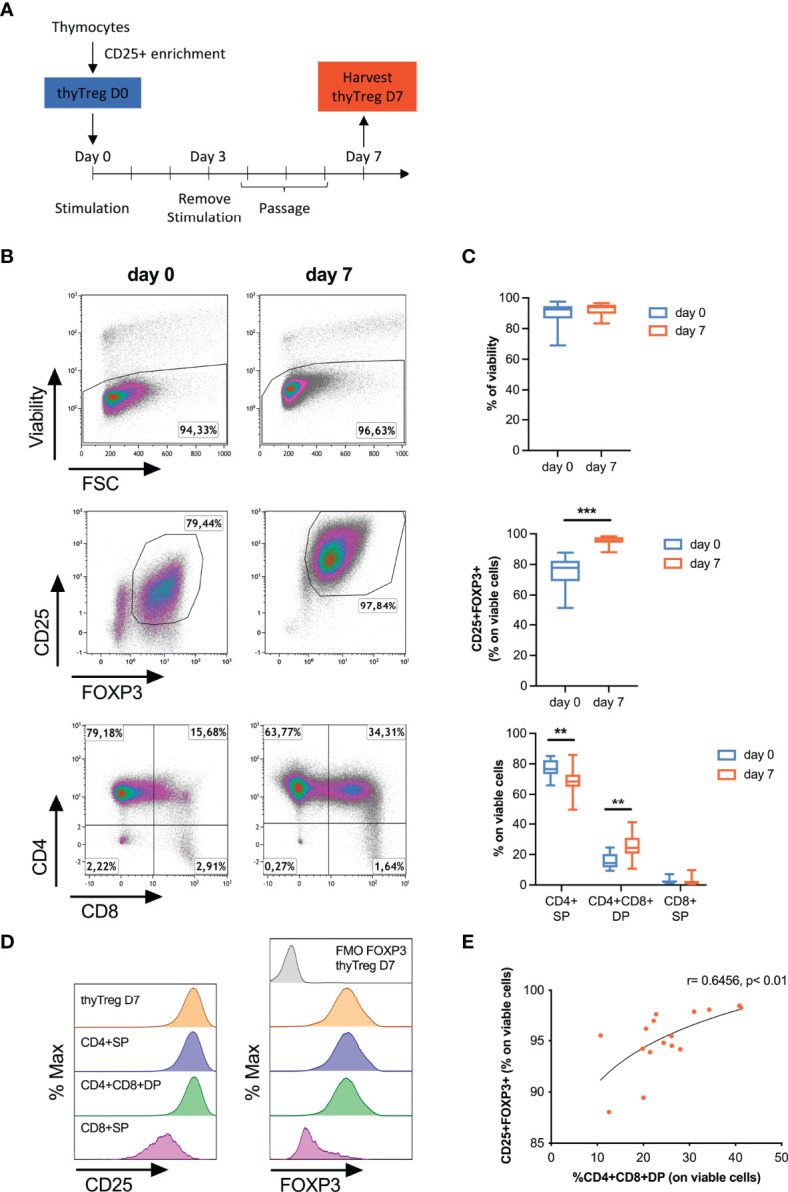
Characteristics of manufactured thyTreg. **(A)** Isolation and culture protocol for thyTreg obtention. **(B)** Representative flow cytometry dot plots showing the viability, purity and CD4/CD8 phenotype of thyTreg right after isolation (day 0) or after culture (day 7). **(C)** Summary of the cell viability, purity and CD4/CD8 phenotype of n=16 thyTreg at days 0 (blue) and 7 (orange). The graph shows min-median-max. **,P < 0.01 and ***,P < 0.001 (paired Wilcoxon test). **(D)** Representative flow cytometry histograms showing CD25 (left) and FOXP3 (right) expression in thyTreg CD4/CD8 subsets. To determine the background signal, the fluorescence minus one (FMO) of FOXP3 is shown. **(E)** Correlation between the frequency of CD4^+^CD8^+^DP and the purity of thyTreg product (Pearson correlation analysis).

Thymic tissue fragments were mechanically disaggregated in TexMACS GMP medium (Miltenyi Biotec) with the gentleMACS Dissociator (Miltenyi Biotec). Total thymocytes obtained were filtered through a 40 µm pore, and CD25^+^ cells were immunomagnetically selected using human CD25 Microbeads II and LS columns (Miltenyi Biotec). After isolation, CD25^+^ (thyTreg day 0) and CD25- (thyTconv day 0) were cultured in TexMACS GMP medium supplemented with 600 U/ml IL-2 (Miltenyi Biotec) at 10^6^ cells/ml at 37°C and 5% CO_2_. Cells were stimulated with T Cell TransAct (Miltenyi Biotec), a polymeric nanomatrix, to activate and expand human T cells *via* CD3 and CD28 following the manufacturer’s instructions. On day 3, half of the medium was removed and replaced with fresh TexMACS GMP medium supplemented with 600 U/ml IL-2. Cells were monitored on days 4, 5 and 6 and passage was performed when required. On day 7, cells were harvested, and their phenotype, functionality and stability were analyzed (**Figure 1A**). Additionally, dried cell pellets and culture supernatants were stored at −80°C for further DNA methylation studies and cytokine quantification respectively.

### GMP thyTreg Manufacturing Protocol in the Cell Production Unit

To implement the thyTreg isolation and culture process in a good manufacturing practice (GMP) compliant protocol, we had to adapt some procedures and reagents as described in **Supplementary Table 1**. The manufacturing process was carried out in the Cell Production Unit (CPU) of Gregorio Marañón Health Research Institute (IISGM), which is accredited by the Spanish Agency of Medicines and Medical Devices (AEMPS). Briefly, thymic fragments were dissociated with the gentleMACS Octo Dissociator equipment (Miltenyi Biotec). Total thymocytes were suspended in PBS/EDTA supplemented with 0.5% human serum albumin (Albutein 20%, Grifols) and poured into a transfer bag (Grifols). CD25^+^ thyTreg were isolated using the CliniMACS CD25 GMP MicroBeads, CliniMACS Tubing Set and CliniMACS plus equipment (Miltenyi Biotec). The positive fraction containing the thyTreg was counted and centrifuged to remove the selection buffer and resuspended in TexMACS GMP medium at 1x10^6^ cells/ml. ThyTreg were cultured in flasks maintaining a concentration of 500,000 cells/cm^2^. After 7 days, the necessary tests were carried out to demonstrate the quality of the thyTreg obtained in the CPU. Both dry cell pellets and cell culture supernatants were stored at −80°C.

Additional sterility analyses were performed during the entire manufacturing process of thyTreg cells at the CPU to confirm the absence of microorganisms. A series of blood cultures were carried out to assess the detection of aerobic and anaerobic microorganisms using the automatic system BACTEC (Beckton Dickinson), in addition to the detection of mycoplasma by bioluminescence in the cell culture supernatant. The detection of genetic abnormalities was performed through an array-CGH (KaryoNIM Stem) by NIMGenetics (Madrid, Spain).

### Flow Cytometry and Cell Sorting

We evaluated the cell viability and phenotype in the different stages of the procedure by flow cytometry. Briefly, cell surface markers staining was followed by staining with Fixable Viability Dye-eFluor450 (eBioscience). Then, the cells were fixed and permeabilized using the FOXP3 transcription factor staining kit (eBioscience) for intracellular staining. All the antibodies are listed in **Supplementary Table 2**. Flow cytometry analysis of labeled cells was performed with a MACSQuant16 cytometer (Miltenyi Biotec), acquiring at least 100,000 events, and the data were analyzed using Kaluza software (Beckman Coulter).

To isolate the CD4^+^ single-positive (SP) and CD4^+^CD8^+^ double-positive (DP) thyTreg cells, 50x10^6^ of total thyTreg cells were labeled with anti-CD4-VioBlue (Miltenyi Biotec) and anti-CD8-FITC (Beckman Coulter). Cells were washed and resuspended at 5x10^6^ cells/ml in MACSQuant Tyto Running Buffer (Miltenyi Biotec) and were subjected to two consecutive rounds of sorting with High-Speed MACSQuant Tyto Cartridges (MACSQuant Tyto cell sorter, Miltenyi Biotec). After the first round, CD4^+^SP cells were collected from the positive fraction. The negative fraction was loaded into a second cartridge, and CD4^+^CD8^+^ DP cells were collected from the positive fraction.

### *In Vitro* Suppression Assay

Peripheral blood mononuclear cells (PBMC) were obtained from buffy coats of healthy donors from the Madrid Transfusion Center and cryopreserved until further use. Thawed PBMC were cultured overnight in RPMI 1640 (Biochrome) supplemented with 5% serum fetal bovine serum (FBS, Biowest) and 60 U/ml of IL-2 (ImmunoTools). The following day, the PBMC were stained with 1 μM of CellTrace Violet (CTVio, Life Technologies). 1x10^5^ CTVio-labeled allogeneic PBMC were co-cultured with thyTreg at different thyTreg : PBMC ratios (1:1, 1:2, 1:4 and 1: 8) in the presence of anti-CD3/anti-CD28 coated-beads (Dynabeads; Gibco) at a bead:PBMC ratio of 0.5:1 in X-VIVO 15 (Lonza) supplemented with 5% serum human AB (Sigma-Aldrich) and 600 U/ml of IL-2 (ImmunoTools) in round bottom 96 well culture plate. PBMC cultured alone in the presence or absence of Dynabeads were used as positive (C+) and negative (C-) control of proliferation, respectively. After 3 days in culture, the cells were labeled with anti-CD4-PC7 (Beckman Coulter), anti-CD8-FITC (Beckman Coulter) and 0.5 µg/mL of 7AAD (Sigma-Aldrich) to differentiate living and dead cells. Cells were acquired in a MACSQuant16 cytometer (Miltenyi Biotec), and data analysis was performed using Kaluza software (Beckman Coulter). The percentage of suppression of proliferation was calculated according to the “Division index method” (29) within CD4^+^ and CD8^+^ T cells.

### Cytokine Production Analysis

The levels of different secreted cytokines or soluble proteins were measured using ELLA Protein-Simple (Biotechne) immunoassay technology in the culture supernatant of the thyTreg product (day 7). The supernatants were thawed at room temperature and centrifuged to remove cell debris. Samples were pre-treated (in case of TGF- β detection) and diluted according to the manufacturer (Simple Plex, Protein Simple). Samples were then loaded along with the necessary controls into SimplePlex cartridges, following the kit instructions for their quantification by triplicate. Graphs show the concentration of each molecule in pg/ml; each point represents the mean of the replicate measurements. The limit of detection (LD) and the quantification range for each of the evaluated molecules are: IL-10, 0.14 (0.46-5530 pg/ml); TGF-β, 5.29 (20.8-12684 pg/ml); Granzyme-B, 0.385 (1.31-5000 pg/ml); LAG-3, 15 (39.6-151050 pg/ml); TIM-3, 0.33 (2.04-7780 pg/ml); IFN-γ, 0.05 (0.17-4000 pg/ml); IL-17A, 0.38 (0.82-8490 pg/ml); IL-4, 0.05 (0.319-1290 pg/ml); and PD-L1, 0.741 (3.45-13172 pg/ml). Values above the limit of quantification are shown as the maximum limit of quantification. Values below the limit LD are shown as 0.

### Stability Assay Under Proinflammatory Conditions

The thyTreg cell product (day 7) was cultured at 1x10^6^ cells/ml in TexMACS GMP medium supplemented with 600 U/ml IL-2 and restimulated with TransAct alone or together with the following cytokines: 10 ng/ml of IL-12 (polarizing condition to Th1); and 10 ng/ml IL-1β, 10 ng/ml IL-6, 10 ng/ml of IL-23 and 20 ng/ml of TNF-α (polarizing condition to Th17). All cytokines were purchased from ImmunoTools. PBMC were also cultured in parallel under the same conditions. Cells were cultured for 3 days, removing excess TransAct matrix on day 2. On day 3, thyTreg and PBMC culture supernatants were frozen at −80°C for cytokine analysis, and thyTreg were assessed for cell viability, phenotype, and suppressive capacity as described above. The remaining cells were saved as dry pellets at −80°C for TSDR methylation studies.

### Methylation Analysis

DNA was isolated from cell pellets using DNeasy Blood & Tissue Kit (Qiagen). The methylation status of 141 CpG sites located in 29 different genome regions comprising 20 different genes, including the Treg-specific demethylated region (TSDR), was analyzed by targeted Next-Gen bisulfite sequencing (NGS070V3 assay) performed by EpigenDx Inc (Hopkinton, MA, USA).

### Statistical Analysis

The results are expressed as the mean ± SEM (Standard Error of the Mean) or min-median-max. Continuous data were tested for normality using the Shapiro-Wilk test. Comparisons were based on the unpaired Mann-Whitney U test and the paired Wilcoxon test for nonparametric data. The statistical test used to evaluate each experiment is specified within the respective figure legend. The statistical associations between variables were calculated by linear regression and Pearson correlation analysis. *p*-values < 0.05 were considered to be statistically significant. The following criteria to distinguish significance levels was used: * = < 0.05, ** = < 0.01 and *** = < 0.001.

## Results

### ThyTreg Isolation and Phenotype

Thymocytes obtained by mechanical disaggregation from freshly removed pediatric thymuses (n=20; age range 0-48 months; **Table 1**) presented high viability (96.33% ± 0.99%) (**Supplementary Figure 1A**). Most of them (76.68% ± 2.03%) exhibited a CD4^+^CD8^+^ double-positive (DP) phenotype, while 12.57% ± 1.23% were CD4^+^ single-positive (SP) cells, and 6.98% ± 1.26% were CD8^+^SP cells (**Supplementary Figure 1B**). Because it has been shown that CD4^+^CD8^+^DP thymic Treg cells significantly contribute to the Treg pool in the human thymus (30–32), we decided to directly isolate CD25^+^ thymocytes (2.36% ± 0.34%) without previous depletion of CD8^+^ cells. The average frequency of FOXP3^+^ cells on isolated CD25^+^ thymocytes was 67.08% ± 2.22% (representative plot in **Supplementary Figure 1C**). The benefits of preserving DP thyTreg were supported by comparing the thyTreg cells obtained with or without CD8^+^ depletion. The thyTreg yield was significantly higher without CD8^+^ depletion (p=0.04; **Supplementary Figure 2A**), as well as the proportion of DP cells (p=0.003; **Supplementary Figure 2B**) while maintaining cell viability and percentages of CD8^+^SP and FOXP3^+^ cells (**Supplementary Figures 2B-D**). Following this strategy, we obtained 6.54 x 10^6^ thyTreg per 10^9^ thymocytes (range 2.44 x 10^6^ – 11.65 x 10^6^) after CD25^+^ immunomagnetic selection (**Table 1**), with cell viability over 85%. Therefore, the estimated thyTreg number per gram of thymus was around 9.96 x 10^6^ (range 1.32 x 10^6^ – 21.59 x 10^6^), which corresponds to 200.3 x 10^6^ highly pure thyTreg for an average thymus weight of 20.10 grams.

**Table 1:** Characteristics of processed thymuses and thyTreg obtention.

Donor ID	Age (mo)	Thymus weight (g)	Thymocytes/g (x 10^9^)	ThyTreg D0 x 10^6^ (per 10^9^ thymocytes)	ThyTreg D7 x 10^6^ (per 10^9^ thymocytes)
1	6	23.51	1.91	7.55	57.08
2	3	36.15	3.37	6.13	18.77
3	8	37.40	1.82	4.29	36.72
4	14	26.17	2.49	8.67	209.47
5	48	15.52	0.76	7.20	40.75
6	0.3	15.20	1.14	11.65	85.65
7	0.1	13.66	1.60	4.90	22.20
8	1	13.24	1.31	8.82	104.96
9	5	34.67	2.11	5.04	14.36
10	0.2	12.00	1.22	7.00	49.18
11	3	12.33	0.88	3.38	4.64
12	0	7.80	1.14	4.75	15.58
13	0.4	5.90	1.21	6.80	23.97
14	30	29.00	1.43	5.33	11.73
15	0.2	3.30	0.95	7.60	30.40
16	0.8	12.16	1.79	8.00	103.20
17*	4	35.20	1.80	9.20	39.65
18*	0.1	12.60	1.11	5.15	56.60
19*	28	47.00	0.54	2.44	20.95
20*	0.8	9.20	1.18	6.85	99.75
**Mean**	7.65	20.10	1.49	6.54	52.28
**Range**	0-48	3.3-47	0.54-3.37	2.44-11.65	4.64-209.47

Individual data, mean and range are shown. *GMP-thyTreg.

### ThyTreg Culture and Product Characterization

Following thyTreg isolation, cells were activated for 3 days and cultured for an additional 4 days, as depicted in **Figure 1A**. It is to note that the culture conditions were kept as simple as possible with the idea of maintaining the immature nature of the thyTreg, avoiding extra compounds usually employed during Treg expansion such as rapamycin and human AB serum, which showed no advantage in terms of thyTreg purity, phenotype or fold expansion (**Supplementary Figures 3A–F**). These cell characteristics were also maintained using TransAct instead of Dynabeads for cell activation to avoid the cell loss associated with the Dynabeads removal (**Supplementary Figures 3G–J**). Cell phenotype on day 0 and day 7 is shown in **Figures 1B, C** (n=16). The thyTreg cells harvested at day 7 presented very high viability (92.41% ± 1.02%) and purity in terms of CD25^+^FOXP3^+^ (95.2% ± 0.74%); being both parameters higher compared to day 0. During this short-time culture period, thyTreg proliferated 6.9 ± 1.42-fold (**Supplementary Figure 4A**). Considering 200.3 x 10^6^ thyTreg isolated at day 0 and the average fold expansion, the theoretical number of thyTreg that could be obtained from a single thymus is around 1,500 x 10^6^, reaching a yield of 13,649 x10^6^ thyTreg cells from a single thymus in the best case.

We observed that the proportion of CD4^+^SP thyTreg decreased during the cell culture, being offset by the increased proportion of CD4^+^CD8^+^DP thyTreg (**Figures 1B, C**; bottom panels**)**. Remarkably, at day 7, these CD4^+^CD8^+^DP thyTreg presented a similar phenotype to the CD4^+^SP thyTreg, characterized by a high expression of CD25 and FOXP3 (**Figure 1D**). Indeed, there was a positive correlation between the proportion of CD4^+^CD8^+^DP thyTreg and the frequency of CD25^+^FOXP3^+^ thyTreg (**Figure 1E**).

**Figure 2:**
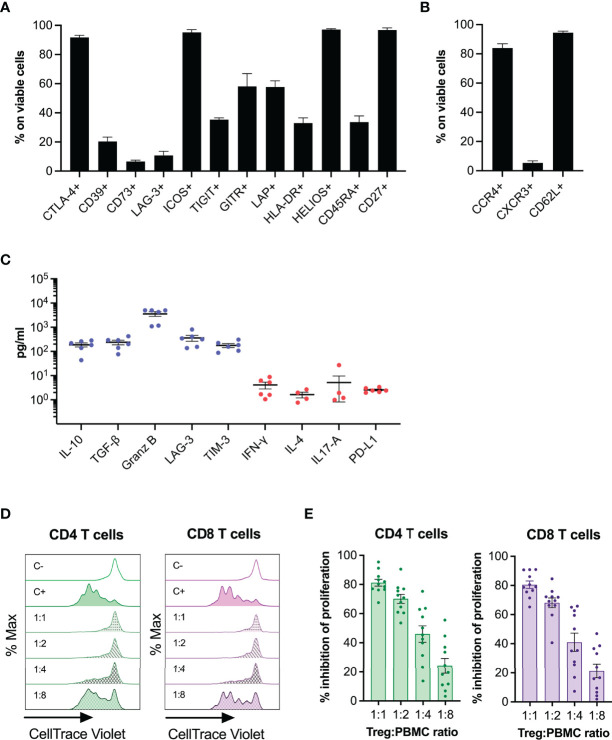
Phenotype and functionality of thyTreg cell product. **(A)** Frequency of phenotypic and functionality markers within thyTreg cells (day 7). **(B)** Frequency of homing markers within thyTreg cells (day 7). **(C)** Quantitation of molecules secreted in day 7 thyTreg culture supernatants. Anti-inflammatory molecules in blue; proinflammatory molecules in red. **(D)** Representative flow cytometry histograms showing CD4 (green) and CD8 T (purple) cell proliferation as CellTrace Violet lost. C-, negative control of proliferation, PBMC cultured alone without stimulation; C+, positive control of proliferation, PBMC cultured alone with anti-CD3/anti-CD28 stimulation; 1:1 to 1:8, stimulated PBMC cultured with thyTreg cells at different thyTreg : PBMC ratios. **(E)** Summary of the suppressive capacity of thyTreg cells defined as % inhibition of CD4 (green) and CD8 T (purple) cell proliferation at the indicated ratios. Graphs show mean ± SEM.

To further characterize the thyTreg product, we analyzed a series of cellular markers related to Treg phenotype and functionality (**Figure 2A**). In summary, thyTreg product was characterized by high expression of the cytotoxic T-lymphocyte associated protein (CTLA-4), inducible T-cell co-stimulator (ICOS), thymic origin marker HELIOS, and CD27; intermediate expression of T cell immunoreceptor with Ig and ITIM domains (TIGIT), glucocorticoid-induced tumor necrosis factor receptor (GITR), latency-associated peptide (LAP), HLA-DR, and CD45RA; and low expression of CD39, CD73, and lymphocyte activation gene 3 (LAG-3). In addition, to evaluate the homing capacity of thyTreg cells, we determined the expression of the chemokine receptors CCR4, CXCR3, and the CD62L selectin (**Figure 2B**). ThyTreg cells showed high expression of CCR4, indicating their putative ability to migrate to organs with large epithelial surfaces (such as skin, gut or lungs) (33), and CD62L, favoring their location in lymph nodes (34). As previously shown (27), the expression of several functionality markers, including CTLA-4, CD73, ICOS, GITR and LAP, significantly increased during the cell culture; whereas CD39 expression decreased (**Supplementary Figure 4B** **)**. Moreover, CD45RA expression increased, which could reflect the last switch from CD45RO to CD45RA occurring as a final step of maturation in the thymus (35) (**Supplementary Figure 4B**). Regarding the expression of chemokine receptors, CCR4 and CD62L significantly increased after 7 days of culture; whereas CXCR3 expression decreased (**Supplementary Figure 4C**), indicative of an undifferentiated phenotype (36).

We then analyzed the profile of secreted molecules by thyTreg in culture supernatants (**Figure 2C**). We detected high levels of the anti-inflammatory cytokines IL-10 and transforming growth factor β (TGF-β) (188.03 ± 36.04 and 237.73 ± 50.83 pg/ml, respectively); as well as high levels of other inhibitory molecules associated with Treg functionality, such as Granzyme B, soluble LAG-3 and soluble T-cell immunoglobulin mucin 3 (TIM3). On the contrary, we detected very low expression of proinflammatory cytokines such as IFN-γ, IL-4, IL-17A; and PD-L1. Finally, we evaluated *in vitro* the capacity of thyTreg cells to suppress the proliferation of CD4^+^, and CD8^+^ stimulated T cells (**Figures 2D, E**). We found that thyTreg exhibited a very high suppressive capacity, with more than 80% mean inhibition at a thyTreg:responder cells ratio of 1:1 and more than 40% at 1:4 ratio.

**Figure 3:**
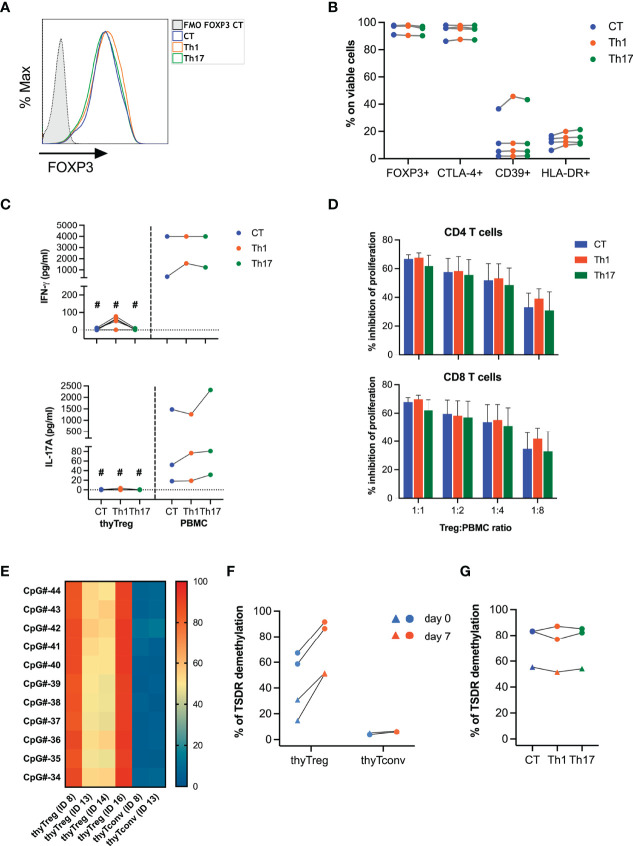
Stability of thyTreg cell product. **(A–D, G)** thyTreg cell product was restimulated under control conditions (CT, blue), or under Th1 (orange) or Th17 (green) polarizing conditions and evaluated after 3 days of culture. PBMC were cultured in parallel under the same conditions. **(A)** Representative flow cytometry histogram showing FOXP3 expression. To determine the background signal, the fluorescence minus one (FMO) of FOXP3 is shown. **(B)** Frequency of FOXP3, CTLA-4, CD39 and HLA-DR within thyTreg under different culture conditions. Paired Wilcoxon test showed no significant differences between conditions. **(C)** Quantitation of secreted IFN-γ and IL-17A by thyTreg or PBMC under different culture conditions. Comparison between culture conditions within the same cell type was performed using paired Wilcoxon test, and comparison within the same condition between thyTreg and PBMC were performed using unpaired Mann-Whitney test (^#^P < 0.05). **(D)** Summary (n=4) of the suppressive capacity of thyTreg cells cultured under different polarizing conditions defined as % inhibition of CD4 (upper panel) and CD8 T (lower panel) cell proliferation at the indicated ratios. Graphs show mean ± SEM. Paired Wilcoxon test showed no significant differences between conditions. **(E)** Demethylation level of 11 conserved CpGs at the TSDR region of *FOXP3* in n=4 thyTreg cell products and n=2 thyTconv cultured in parallel for 7 days. ID13 and ID14 are female donors. **(F)** Global TSDR demethylation level (calculated as the mean of demethylation of the 11 CpGs) of thyTreg and ThyTconv right after cell isolation (day 0, blue) or after 7 days of culture (day 7, orange). Triangles represent female donors, and circles represent male donors. **(G)** Global TSDR demethylation level of thyTreg cultured under different polarizing conditions.

To determine the stability of the thyTreg product, we restimulated thyTreg cells exposed to a cocktail of cytokines polarizing to Th1 (IL-2, IL-12) or polarizing to Th17 (IL-2, IL-1β, IL-6, IL-23, TNF-α). We observed that thyTreg cell phenotype in terms of FOXP3, CTLA-4, CD39, and HLA-DR expression remains imperturbable (**Figures 3A, B**). Furthermore, thyTreg cells were not prompted to produce IFN-γ or IL-17A under polarizing conditions (**Figure 3C**) and conserved their suppressive function (**Figure 3D**). To support these findings, we determined the stability of *FOXP3* expression by analyzing the methylation profile of the TSDR (**Figures 3E, F**). We observed an intermediate level of TSDR demethylation in thyTreg cells at day 0 (62.93% ± 4.3% for males and 22.74% ± 8.03% for females), which increased after culture to 89.03% ± 2.57% for males and 51.26% ± 0.36% for females. Differences in demethylation levels between gender is due to the methylation-mediated inactivation of one X-chromosome in females. In contrast, the TSDR demethylation of freshly isolated (day 0) or cultured (day 7) thymic CD25- (thyTconv) was around 5%. The differential methylation pattern between thyTreg and thyTconv was observed not only in the *FOXP3* gene but in other 8 out of 19 genes related to Treg, including *CTLA-4*, *IKZF2* or *ILR2A* (**Supplementary Figure 4D** **)**. Notably, the TSDR demethylation status in thyTreg cells was maintained under proinflammatory conditions (**Figure 3G**).

Since one of the hallmarks of our thyTreg product is the existence of a CD25^+^FOXP3^+^CD4^+^CD8^+^ DP population, we decided to evaluate their commitment to a Treg phenotype by analyzing the methylation status of the TSDR. For that, after 7 days of thyTreg culture, we sorted the CD4^+^SP and the CD4^+^CD8^+^DP populations and analyzed their TSDR demethylation status compared with the whole thyTreg cell product (**Supplementary Figure 5**). We indeed confirmed that the proportion of CD4^+^CD8^+^DP cells with a demethylated TSDR (94.1%) was similar to that observed in the CD4^+^SP or the total thyTreg population (92.8% and 91.6%, respectively), confirming the stability of *FOXP3* expression in this cell subset.

### GMP thyTreg

**Figure 4:**
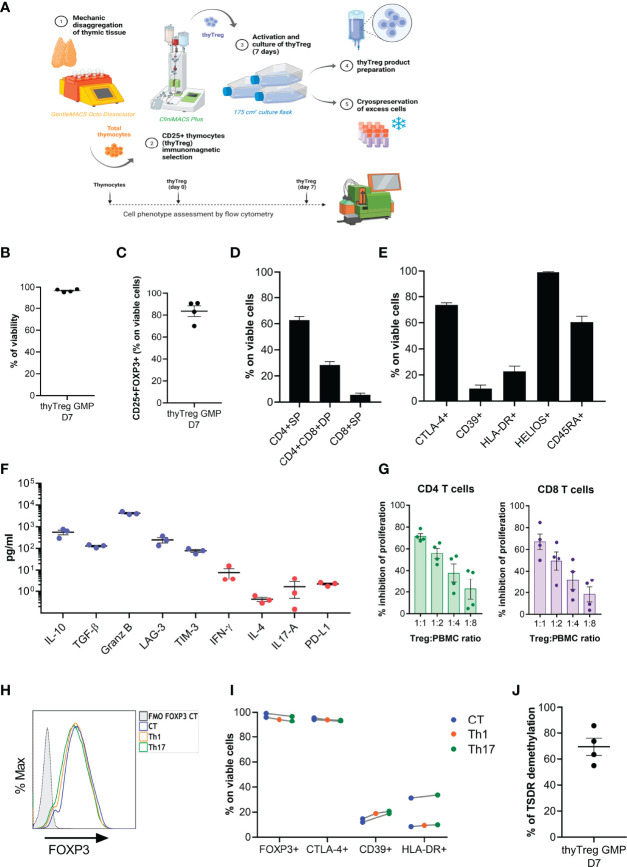
GMP manufacturing of thyTreg. **(A)** Schematic representation of the procedure and equipment used in the Cell Production Unit for the thyTreg GMP manufacturing. Additionally, the dotted line represents the scheme of the quality evaluation process performed at different stages. **(B–E)** Summary of the cell viability **(B)**, purity **(C)** and phenotype **(D, E)** of n=4 GMP thyTreg. Graphs show mean ± SEM. **(F)** Quantitation of molecules secreted in day 7 GMP thyTreg culture supernatants. Anti-inflammatory molecules in blue; proinflammatory molecules in red. **(G)** Summary of the suppressive capacity of GMP thyTreg cells defined as % inhibition of CD4 (green) and CD8 T (purple) cell proliferation at the indicated ratios. Graphs show mean ± SEM. **(H)** Representative flow cytometry histogram showing the stability of FOXP3 expression of GMP thyTreg cell product under control conditions (CT, blue) or under Th1 (orange) or Th17 (green) polarizing conditions evaluated after 3 days after re-stimulation. To determine the background signal, the fluorescence minus one (FMO) of FOXP3 is shown. **(I)** Frequency of FOXP3, CTLA-4, CD39 and HLA-DR within GMP thyTreg under different polarizing conditions. **(J)** Global TSDR demethylation level (calculated as the mean of demethylation of the 11 CpGs) of GMP thyTreg cell product (n=4 males).

With a focus on the possible therapeutic use of thyTreg cells, all the manufacturing protocol carried out at the research level was made considering that the reagents and equipment used were GMP-compliant or that GMP-certified equivalents were available in the market (**Supplementary Table 1** and **Figure 4A** **)**. To confirm that thyTreg cells presented the same quality and functionality under GMP conditions, four thyTreg cell products (mean age of thymus donor = 8.23 months, **Table 1**) were manufactured at the Cell Production Unit (CPU) of Gregorio Marañón Health Research Institute.

Following thymic dissociation, the recovery of thyTreg cells after the CD25^+^ selection with the CliniMACS was around 5.91 x 10^6^ per 10^9^ labeled thymocytes, a value that was similar to that obtained using the column selection in the laboratory (6.69 x 10^6^ per 10^9^ labeled thymocytes). GMP-thyTreg cell products obtained in the CPU after 7 days of culture showed a very similar quality to the thyTreg obtained in the laboratory (**Table 2**). The results in **Table 2** show that the GMP-thyTreg products have high viability and purity (**Figures 4B, C**), comparable to laboratory thyTreg. The GMP-thyTreg proliferation in culture was similar to that obtained in the laboratory (**Supplementary Figure 6A**). The GMP-thyTreg cell product presented a similar phenotype, in terms of CD4^+^SP and CD4^+^CD8^+^ DP abundance and other maturation and functionality markers (**Figures 4D, E**). It is to note that the expression of CTLA-4 and CD39 in the GMP-thyTreg cell products was slightly lower than the results obtained in the laboratory thyTreg. Still, their values fall within the ranges observed in laboratory thyTreg cells and are comparable to those of other studies (27). They also secreted a similar pattern of modulatory molecules: high levels of IL-10 (560.33 ± 137.56 pg/ml), TGF-β (129 ± 12.1 pg/ml), Granzyme B, soluble LAG-3 and soluble TIM3; and very low amounts of the proinflammatory cytokines IFN-γ, IL-4, IL-17A; and PD-L1 (**Figure 4F**). In accordance, GMP-thyTreg cells maintained a high suppressive capacity, inhibiting CD4^+^ and CD8^+^ T cell proliferation (**Figure 4G**). Furthermore, the GMP-thyTreg product was stable under proinflammatory Th1 and Th17 conditions, maintaining its phenotype (**Figures 4H, I**), without overexpressing IFN-γ (Th1) or IL-17A (Th17) (**Supplementary Figure 6B** **),** and its functionality remains unchanged (**Supplementary Figure 6C** **)**. They also presented a high percentage of TSDR demethylation, indicative of the stability of the *FOXP3* expression (**Figure 4J**). Additionally, thyTreg cells met all the safety criteria for cell therapy liberation in terms of absence of contaminants and lack of genomic abnormalities.

**Table 2:** Comparison of the main thyTreg characteristics between Research and GMP manufacturing protocol.

Characteristic	GMP thyTreg (n=4)	ThyTreg (n=16)
Donor age (mo)	8.23 ± 6,65	7.50 ± 3.32
Thymocytes/g (x 10^9^)	1.16 ± 0.26	1.57 ± 0.17
thyTreg D0 (per 10^9^ thymocytes)	5.91 ± 1.42	6.69 ± 0.52
thyTreg D7 (per 10^9^ thymocytes)	54.24 ± 16.83	51.79 ± 13.17
**thyTreg D7 phenotype**		
% of Viability	96.58 ± 0.71	92.41 ± 1.02
% of Purity (CD25^+^FoxP3^+^)	83.65 ± 4.87	95.20 ± 0.74
% of CD4^+^SP	62.81 ± 2.70	67.73 ± 2.30
% of CD4^+^CD8^+^DP	28.43 ± 2.59	25.49 ± 2.26
% of CTLA-4^+^	73.71 ± 1.55	91.68 ± 1.53
% of CD39^+^	9.64 ± 2.68	20.39 ± 3.00
% of HLA-DR^+^	22.76 ± 4.00	32.92 ± 3.58
IL10 secretion (pg/ml)	560.33 ± 137.56	188.03 ± 36.04
% of Inhibition of T CD4 proliferation (1:1)	71.48 ± 2.52	81.24 ± 2.31
% of Inhibition of T CD8 proliferation (1:1)	66.96 ± 7.16	80.52 ± 2.54
TSDR demethylation (in males)*	68.08 ± 9.15	89.03 ± 2.57

*Demethylation data corresponds to n=4 for GMP thyTreg and n=2 for research thyTreg. Data are mean ± SEM.

In summary, the adaptation and scaling of the protocol to GMP conditions with the validations performed, provided evidence that we are manufacturing a product of thyTreg cells complying with the specifications required for their use in humans. In fact, our therapeutical product defined as “Treg lymphocytic cells, autologous, obtained from thymic tissue, expanded and stimulated with IL-2 (thyTreg)” received the approval of the Spanish Medical Agency (AEMPS) to be employed as cell therapy in humans.

## Discussion

The existing scientific evidence points to the fact that only through the induction of immunological tolerance we will be able to overcome harmful immune responses, eliminating the use of pharmacological immunosuppression, thus avoiding the toxic effects of these therapies and maintaining a competent immune system (15, 37). Among the different cell-based therapy strategies aiming at this purpose, Treg cells have been shown to play a crucial role in restoring immunological balance (15, 22, 38). In the context of solid organ transplantation, numerous clinical trials have been conducted in adults using autologous therapy of Treg cells obtained from peripheral blood to prevent solid organ rejection (21). However, very few have published efficacy results in its use as a therapy to prevent transplant rejection. One of the pioneers employing therapeutic Treg is the international consortium “The ONE Study”, which administered peripheral blood autologous Treg in adult kidney transplant recipients. Published results proved the feasibility and safety of autologous Treg administration and showed that, while rejection rates were not modified in the first year, Treg infusion was associated with a lower incidence of infections, compared with the reference group (23).

The therapeutic use of *ex vivo* expanded peripheral blood Treg has presented a series of limitations that have compromised its effectiveness. The Treg frequency in peripheral blood is only 5-10% of CD4 T cells (39), therefore the maximum amount of Treg that could be obtained from an adult is around 30 million. Moreover, most peripheral blood adult Treg cells present a memory phenotype (CD45RA−), which indicates a higher phenotypic instability due to a more methylated status of the *FOXP3* and limited suppressive capacity (40). Also, shortened telomers of adult blood-derived Treg affect their replicative potential and *in vivo* survival, limiting the duration of the therapeutic effect (41, 42). This limited quality of adult Treg is worsened due to the long expansion rounds required to reach a sufficient number of Treg for therapeutic use, causing a more senescent phenotype, a marked loss of suppressive capacity and even the conversion of Treg into effector cells that could pose an added risk of rejection (24, 43). In pediatric subjects, Treg cells exhibit a predominantly naïve phenotype with still immature cells that have not been exposed to marked activation and differentiation processes. Several authors have confirmed the higher quality of the naïve Treg (40), indicating that the population of CD45RA^+^ Treg cells, more abundant in children, would be the most appropriate to expand for therapeutic purposes (44, 45). Indeed, therapeutic Treg isolation strategies, including CD45RA+ enrichment, allow to obtain a cell population that maintains its suppressive properties and effectiveness longer (46). Despite the high quality of pediatric Treg, the usual strategy of purifying them from peripheral blood would be unapproachable due to the low blood volume that could be drawn from pediatric subjects, being the maximum amount of recovered Treg around 5 million cells. This could be solved by *ex vivo* expansion cycles, but it would lead to the loss of their undifferentiated phenotype. Another successful strategy is the obtention of Treg from umbilical cord blood (47). These cells share the advantage of a mostly naïve phenotype but also has limitation in the number of cells that can be recovered. As described in Riley et al., 5-7 million Treg (26) can be obtained from a cord blood unit, which would still be a deficient number for a therapeutic dose, requiring therefore numerous rounds of expansion. Indeed, in some cases, it was necessary to expand up to 27,000 times to get a single therapeutic dose (18, 48). Despite the limitation in the number of cells available, the potential efficacy of cord blood Treg has been demonstrated by the excellent results obtained when using them as allogeneic therapy in the prevention of GVHD in adults, reducing the incidence of grade II-IV acute GVHD and eliminating the incidence of chronic GVHD (17, 18).

In a further attempt to improve the therapeutic Treg quality and overcome the current limitations regarding cell number and phenotype, the thymus, a primary lymphoid organ where the T cells mature, has been proposed as a new source of Treg. Indeed, Dijke and collaborators (27) showed that a large amount of stable, long-lived and potent FOXP3^+^ Treg could be isolated and expanded from a single thymus. Furthermore, Romano and colleagues (28) have recently reported a good manufacturing practice (GMP) compliant protocol to isolate and expand thymus-derived Treg cells, confirming the feasibility of the strategy. This is a revolutionary approach since children with heart diseases requiring cardiac surgery often undergo thymectomy to clear the surgical field. Therefore, the thymus is routinely discarded and could provide an excellent source for therapeutic Treg (as an example, around 100 thymuses are discarded per year at our institution, the Pediatric Hospital Gregorio Marañón).

Our thyTreg manufacturing protocol also employs the thymus as a Treg source, but differentiates from the others in several aspects. First, the Treg purification procedure is performed in a single step (immunomagnetic selection of CD25^+^ cells), without previous depletion of CD8^+^ cells. In addition to increasing cell yield, this alternative preserves a CD25^+^ population which is CD4^+^CD8^+^ double-positive (thyTreg DP), for which epigenetic and transcriptional analysis have demonstrated their Treg commitment (32). Moreover, their immature phenotype and high expression of FOXP3 that we observed in thyTreg DP could contribute to the greater purity and suppressive capacity in the final product. Second, we replaced the mechanism of activation used in the other strategies, which are magnetic spheres coupled to anti-CD3/anti-CD28, with a new soluble nanomatrix system that can be easily removed by centrifugation, preventing the loss of a large part of cells in the process of elimination of the spheres prior to administration. Third, the culture medium has been kept to minimum components (TexMACS + human IL-2), avoiding the use of chemical compounds (such as rapamycin) or human sera. Finally, the culture duration is very short (7 days), allowing the activation and proliferation of thyTreg, but avoiding extensive expansion rounds that could potentially decrease the quality of Treg. The thyTreg product obtained exhibits high purity and suppressive capacity, with a stable FOXP3 expression, whose characteristics are maintained under inflammatory conditions. Regarding the number of therapeutic Treg cells obtained, there is a wide variety of product yields, functionality and fold changes depending on the cell source and the protocol employed to isolate and culture Treg (3, 49, 50). Considering the T-cell receptor (TCR) repertoire, both thymic and peripheral Treg have been shown to present very diverse TCR repertoires (51, 52). We acknowledge that Treg isolated from the thymus could potentially present different maturation statuses and indeed be subjected to a partial thymic selection. Nevertheless, most FOXP3^+^ thymocytes are found in the thymic medulla (53), where cells have already been selected. Furthermore, the thyTreg product has been cultured for 7 days and underwent a final step maturation as suggested by the phenotypic marker’s evolution CD45RA, CD62L and CD39. Nonetheless, we are planning to evaluate the TCR diversity of the thyTreg product. Although numerous studies reflect the difficulty of freezing Treg cells while preserving their phenotype and suppressive capacity (54), preliminary data indicates that our thyTreg product can be cryopreserved under a GMP compliant protocol capable of maintaining the viability, phenotype and functionality of the thyTreg, which would make the use of frozen cells feasible. Nevertheless, further investigation in this line is being performed in order to have conclusive results.

The loss of immunological homeostasis and the appearance of excessive or unwanted immune responses in the form of inflammatory phenomena can trigger various serious pathologies. The improved quality and amount of thyTreg obtained with our protocol allow us to prepare hundreds of therapeutic doses from a single thymus, which can be cryopreserved and could be employed for sequential autologous doses or as an “off-the-shelf” allogeneic therapy in another individual. Although the autologous application of thyTreg could be the most straightforward, we postulate that their allogeneic use would be a realistic approach, opening the possibility to treat other diseases, both in children and adults, such as the rejection of different types of organs, GVHD, autoimmune processes, or even in the most severe COVID-19 patients. For all these reasons, we are currently exploring whether the administration of allogeneic thyTreg will maintain its therapeutic suppressor effect without being recognized as foreign and rejected by the recipient´s immune system. This hypothesis is based on preliminary results of our group and others (55) that indicate that the immature or undifferentiated character of the thyTreg is associated with a very low frequency of immunogenicity markers that allow them to be recognized as foreign cells, therefore, being unnoticed by the recipient’s immune system. Indeed, there are already different successful studies that use allogeneic Treg in the context of GVHD employing donor peripheral blood (56) or third-party donor umbilical cord blood (18, 48). However, until the low immunogenicity of our thyTreg product is completely proved, we should consider the importance of HLA-concordance to prevent rejection when using them allogeneically. In addition to the allogenic use of thyTreg, we are also exploring their genetic modification to enhance their effectiveness and versatility. In particular, we are genetically modifying thyTreg to make them antigen-specific by inducing the expression of the Chimeric Antigen Receptor (CAR) and universal by eliminating the HLA from the surface of the CAR-thyTreg.

Importantly, our thyTreg product has been approved by the Spanish Drug Agency (AEMPS) to be administered as cell therapy, and we are recruiting patients in a phase I/II clinical trial that evaluates the safety and efficacy of autologous thyTreg administration to prevent rejection in heart transplant children (NCT04924491). Our ongoing clinical trial, with four patients already treated, is the first to employ a Treg therapy to prevent rejection in transplanted children, but above all, it is the first worldwide to use thyTreg in humans as an alternative to Treg obtained from blood. The confirmation in this trial of the feasibility and safety of our strategy paves the way for the development of new indications for this therapy, which could revolutionize the treatment of different pathologies with high incidence.

## Data Availability Statement

The raw data supporting the conclusions of this article will be made available by the authors, without undue reservation.

## Ethics Statement

The studies involving human participants were reviewed and approved by Ethical Committee (CEIM) from Gregorio Marañon University Hospital. Written informed consent to participate in this study was provided by the participants’ legal guardian/next of kin.

## Author Contributions

EB-Q and MM-B designed and performed experiments, and analyzed data and wrote the manuscript. BC and RL performed experiments and analyzed data. JG-J, CP, AP, and RP-C provided samples of thymic tissue. MCa and NG participated in the enrolment of patients. MCl, MF-S, and SS provided scientific input and support in implementing the manufacturing protocol in the Cell Production Unit. MP and RC-R conceptualized the study, supervised the project and wrote the manuscript. All authors contributed to the article and approved the submitted version.

## Funding

This work was supported by grants from “Fundación Familia Alonso” (FFA-FIBHGM 2019), Instituto de Salud Carlos III (ISCIII) co-financed by FEDER funds (ICI20/00063; PI21/00189; PI18/00495; PI18/00506). EB-Q was supported by a grant from Comunidad de Madrid (EXOHEP-CM. B2017/BMD3727). MM-B was supported by the Sara Borrell Program from ISCIII (CD18/00105) and Marie Sklodowska-Curie program from H2020 (MSCA-IF-EF-RI. 101028834).

## Conflict of Interest

The authors declare that the research was conducted in the absence of any commercial or financial relationships that could be construed as a potential conflict of interest.

## Publisher’s Note

All claims expressed in this article are solely those of the authors and do not necessarily represent those of their affiliated organizations, or those of the publisher, the editors and the reviewers. Any product that may be evaluated in this article, or claim that may be made by its manufacturer, is not guaranteed or endorsed by the publisher.
