# Supplementary material for: A Novel GMP Protocol to Produce High-Quality Treg Cells From the Pediatric Thymic Tissue to Be Employed as Cellular Therapy
Source: Front Immunol. 2022 May 16;13:893576. doi: 10.3389/fimmu.2022.893576 (PMC9148974; doi:10.3389/fimmu.2022.893576)
Supplement: Supplementary file 1 [file DataSheet_1.docx]

Supplementary Material

## SUPPLEMENTARY FIGURES


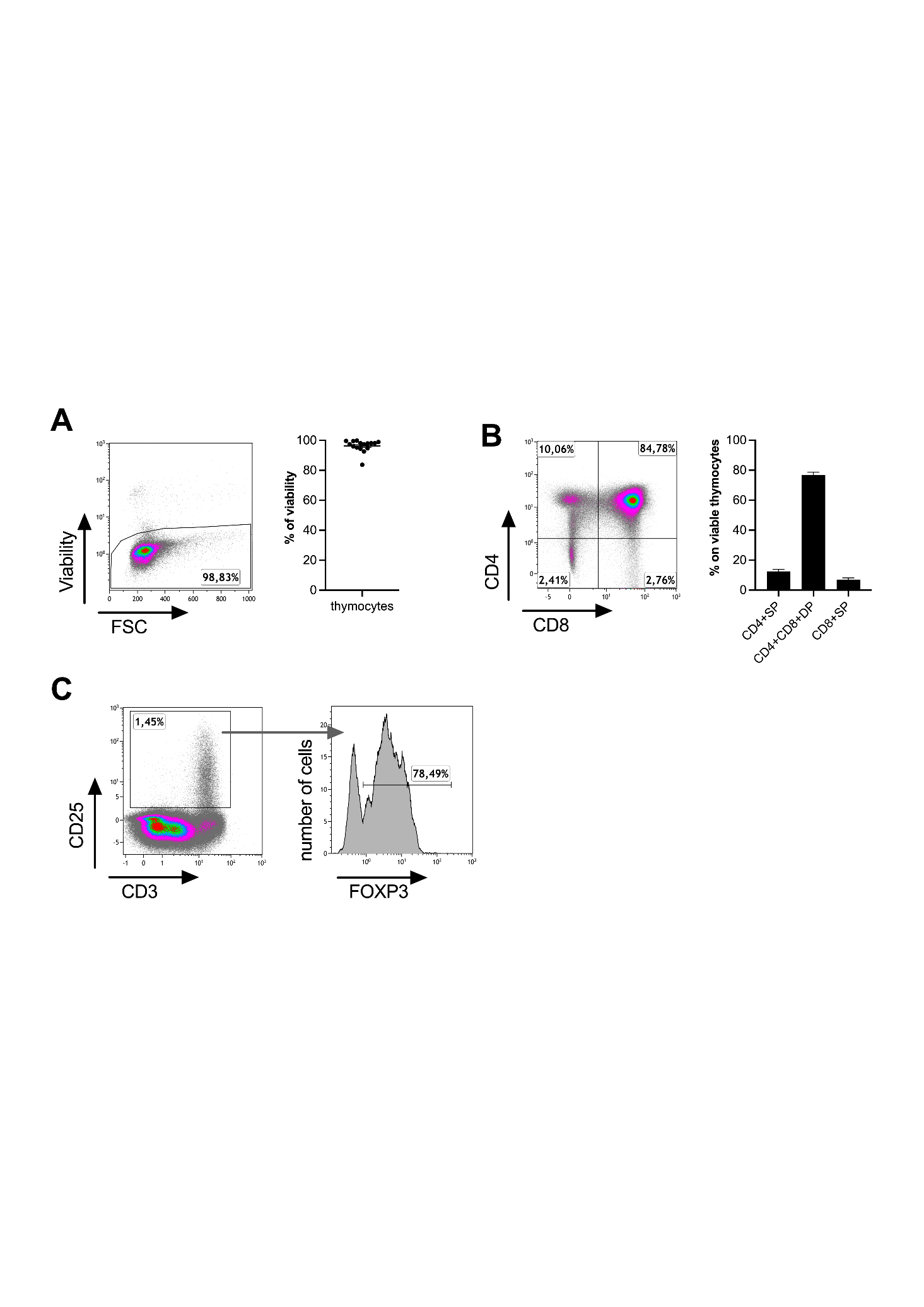


**Supplementary Figure 1. Obtention and characterization of thymocytes from human thymuses. (A)** Representative flow cytometry plot and summary data of thymocytes viability after mechanical dissociation of thymic tissue. **(B)** Representative flow cytometry plot and summary of the CD4/CD8 phenotype of thymocytes. **(C)** Representative flow cytometry plot showing the frequency of CD25+ thymocytes and the expression of FOXP3 within CD25+ thymocytes. Graphs show mean ± SEM.


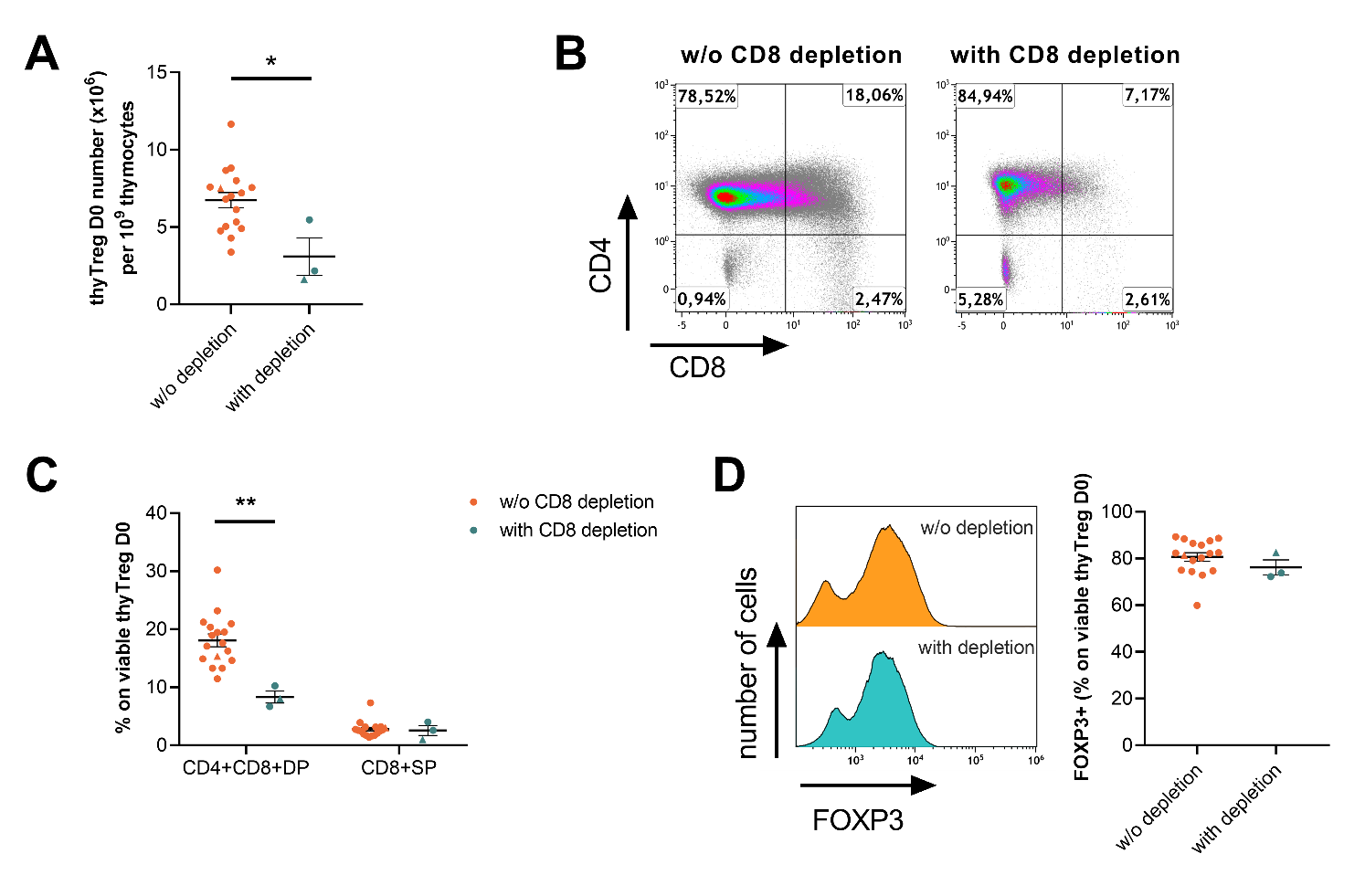


**Supplementary Figure 2. Comparison of freshly isolated thyTreg with or without previous CD8 depletion.** We compared thyTreg cells obtained using the standard protocol (without previous CD8 depletion, w/o depletion, n=17; orange) or with an extra step of CD8 depletion employing the complement-mediated lysis technique with anti-CD8a (OKT-8) and rabbit complement HLA-ABC before CD25+ selection (n=3, green). **(A)** Yield of thyTreg obtained at day 0. **(B)** Representative flow cytometry plots and **(C)** summary of thyTreg CD4/CD8 phenotype with or without CD8 depletion. **(D)** Representative flow cytometry histograms and summary data of FOXP3 expression frequencies within isolated thyTreg on day 0. Graphs show mean ± SEM**.** Comparison between strategies was made using unpaired Mann-Whitney test; *, P < 0.05; and **, P < 0.01. Triangle symbol represents the thyTreg cell products obtained in parallel with both strategies from the same thymic tissue.


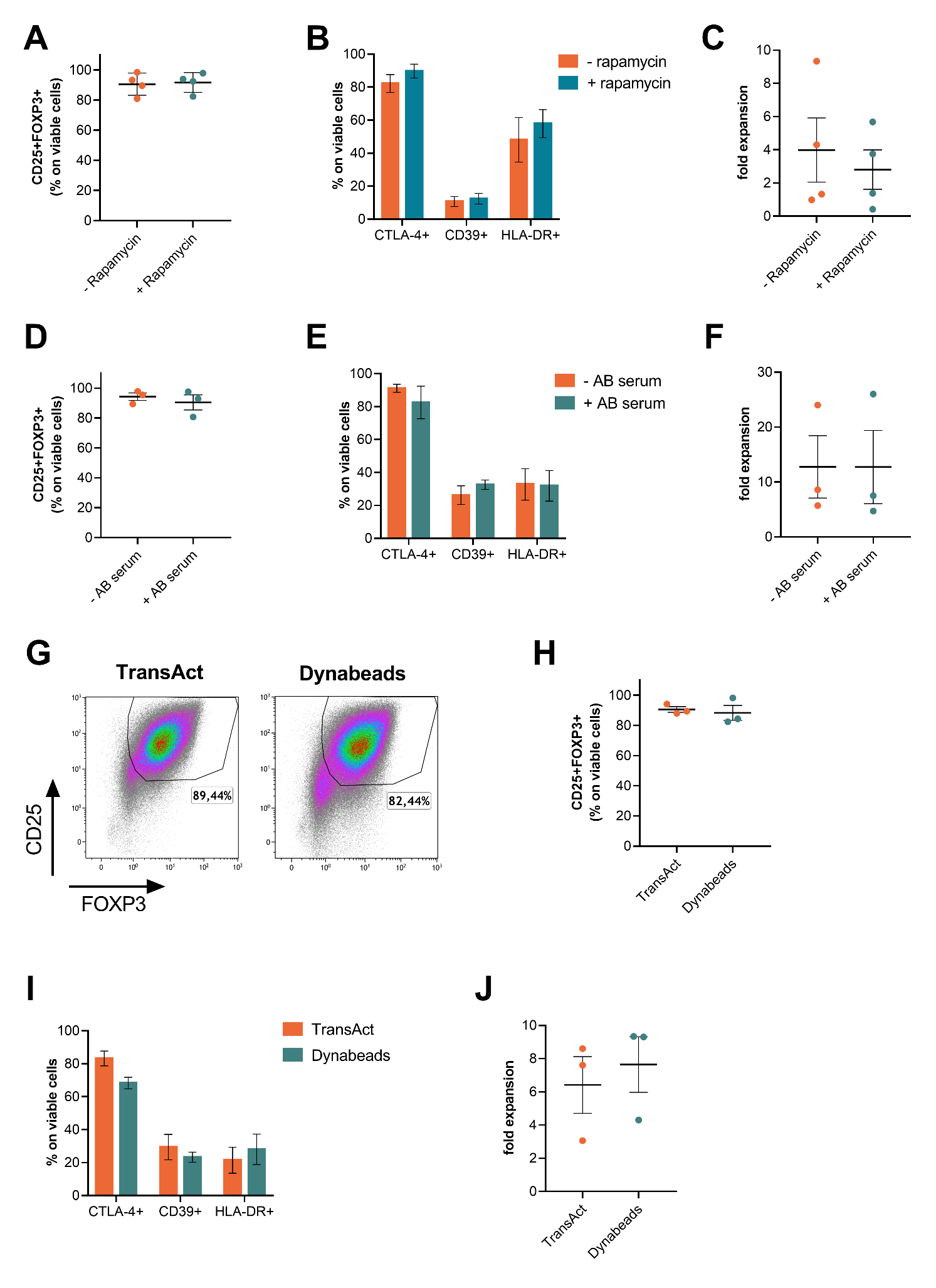


**Supplementary Figure 3. Optimization of thyTreg culture conditions.** Freshly isolated thyTreg were cultured in parallel under the standard conditions (orange) or under the test condition (green), and their phenotype was evaluated at day 7. **(A)** Purity, **(B)** phenotype, and **(C)** fold expansion of thyTreg cultured without or with rapamycin at 50 nM final concentration (n=4). **(D)** Purity, **(E)** phenotype, and **(F)** fold expansion of thyTreg cultured without or with human 5% AB serum (n=3). **(G)** Representative flow cytometry dot plots and **(H) s**ummary of purity, **(I)** phenotype and **(J)** fold expansion of thyTreg stimulated with TransAct or with Dynabeads at ratio 1:1 (n=3). Graphs show mean ± SEM**.** We determined no significant differences between cultured conditions by paired Wilcoxon test.


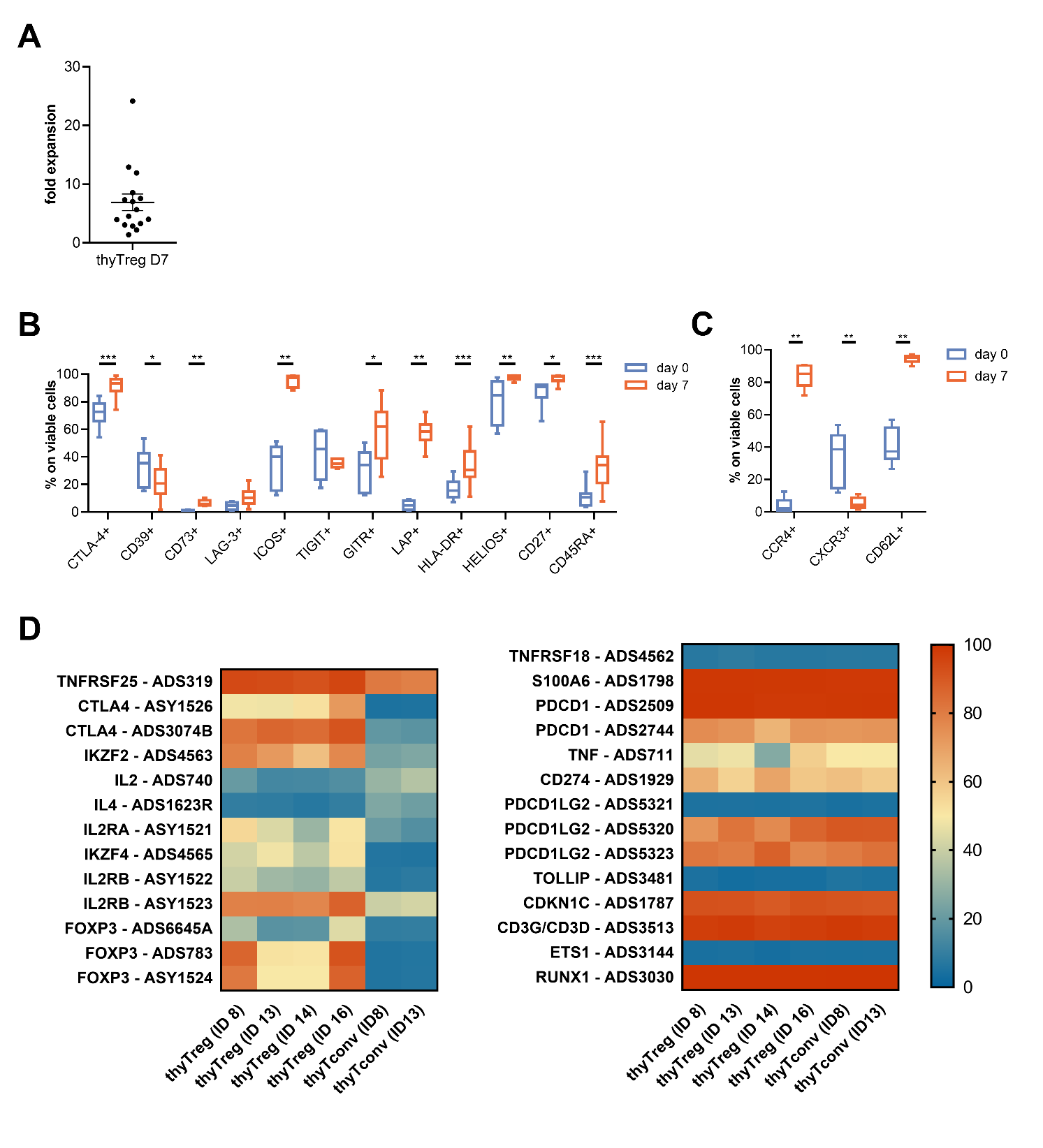
**Supplementary Figure 4. Additional thyTreg characterization. (A)** Fold expansion of thyTreg cells over the manufacturing protocol, n=16 (mean ± SEM). **(B, C)** Summary of the evolution in phenotypic and functionality markers expression within thyTreg cells between day 0 (blue) and day 7 (orange). Graphs show min-median-max. *, P < 0.05; **, P < 0.01; and ***, P < 0.001 (unpaired Mann-Whitney test). **(D)** Global demethylation level of 27 genome regions located in 20 genes (calculated as the mean of demethylation of the CpGs contained in the region) within n=4 thyTreg cell products and n=2 thyTconv cultured in parallel for 7 days. ID13 and ID14 are female donors. The left panel depicts regions with different demethylation patterns within thyTreg and thyTconv. The right panel shows regions with similar demethylation patterns within thyTreg and thyTconv.


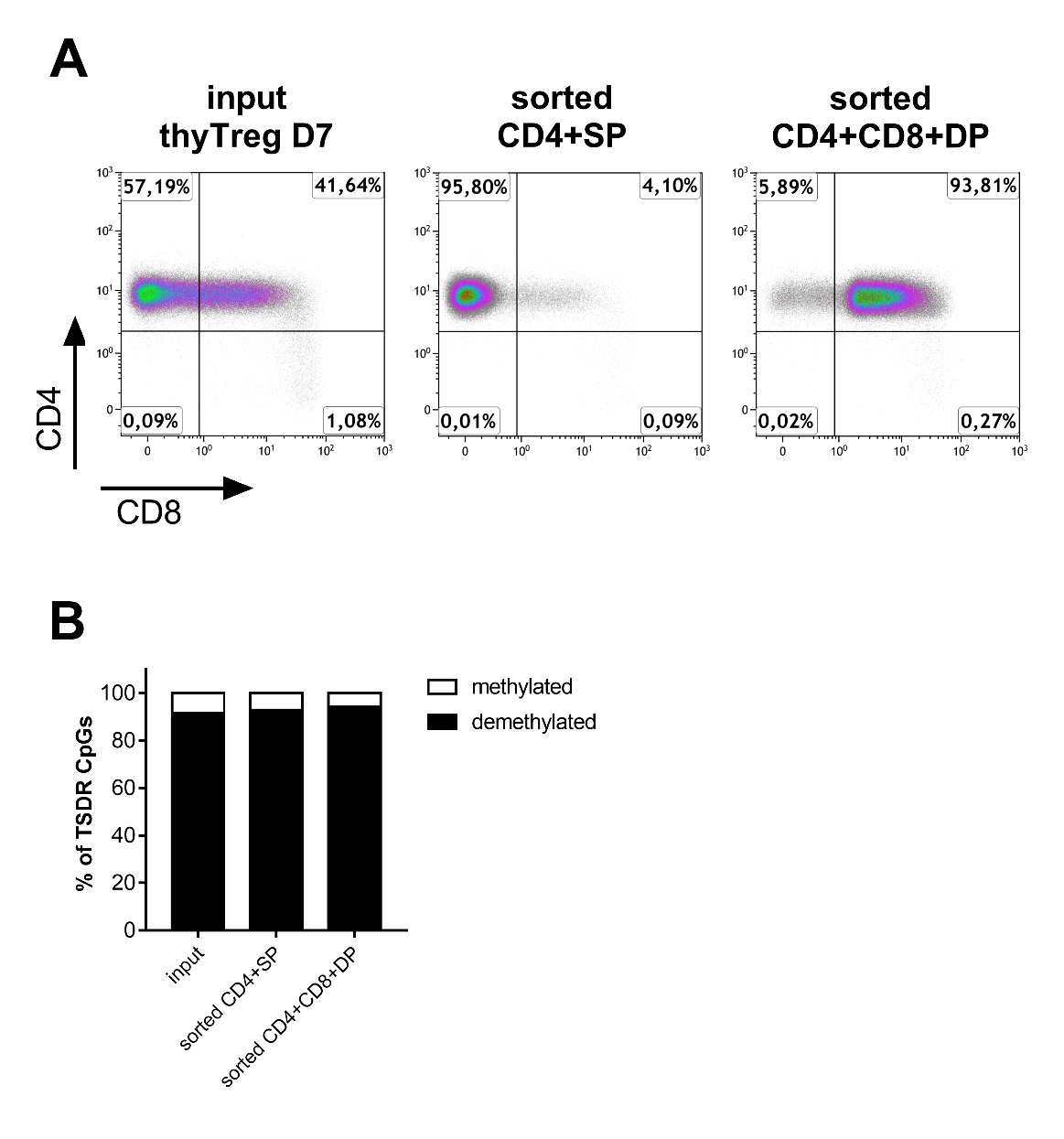


**Supplementary Figure 5. TSDR methylation pattern of total thyTreg, sorted CD4+SP and sorted CD4+CD8+DP. (A)** Flow cytometry plots show the CD4/CD8 phenotype of one thyTreg product before (input) and after sorting of CD4+SP and CD4+CD8+DP subpopulations. The purity of sorted fractions is above 90%. **(B)** Percentage of methylated and demethylated CpG islands within the *FOXP3* TSDR of the input and sorted fractions.


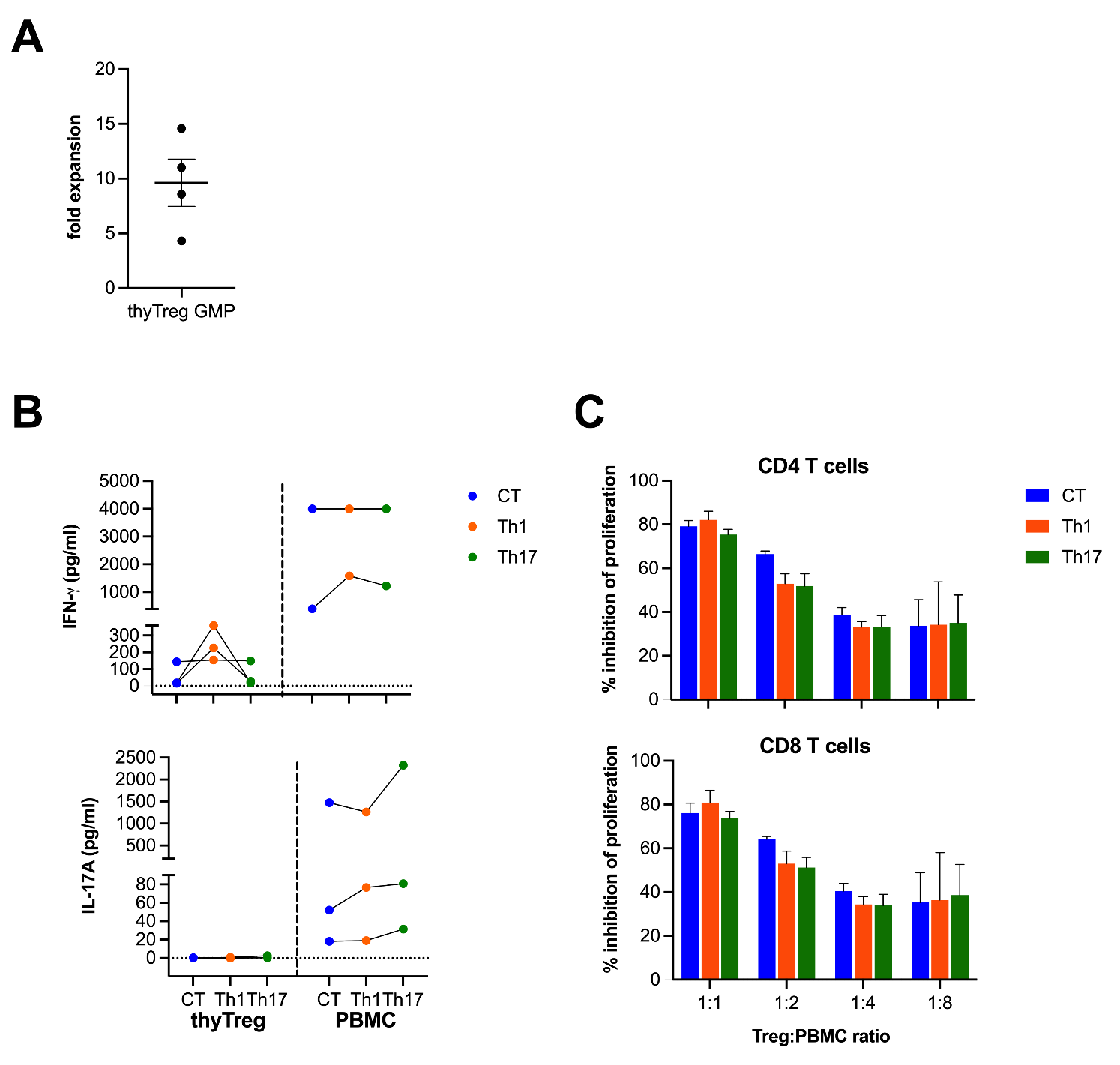


**Supplementary Figure 6. Additional GMP-thyTreg characterization. (A)** Fold expansion of thyTreg cells during the GMP-manufacturing protocol, n=4 (mean ± SEM). **(B)** Quantitation of secreted IFN-γ and IL-17A by GMP thyTreg after 3 days of re-stimulation under control conditions (CT, blue), Th1 (orange) or Th17 (green) polarizing conditions. PBMC were cultured in parallel under the same conditions. When permited, comparisons between culture conditions within the same cell type were performed using paired Wilcoxon test, and comparisons within the same condition between thyTreg and PBMC were performed using unpaired Mann-Whitney test. **(C)** Summary of the suppressive capacity of GMP thyTreg cells cultured under different polarizing conditions defined as % inhibition of CD4 (upper panel) and CD8 T (lower panel) cell proliferation at the indicated ratios. Graphs show mean ± SEM. Paired Wilcoxon test showed no significant differences between conditions.

## SUPPLEMENTARY TABLES

|  | **Research** | **GMP** | **Source** |
| --- | --- | --- | --- |
| **Tissue Dissociation** |  |  |  |
| Dissociator | GentleMACS Dissociator | GentleMACS Octo Dissociator | Miltenyi Biotec |
| **CD25+ Selection** |  |  |  |
| Magnetic cell separator | QuadroMACS Separator and LS columns | CliniMACS plus System and tubing Set | Miltenyi Biotec |
| Selection Reagent | CD25 Microbeads II | CliniMACS CD25 Reagent | Miltenyi Biotec |
| Selection Buffer | AutoMACS Running Buffer | CliniMACS PBS/EDTA Buffer | Miltenyi Biotec |
| **Cell culture** |  |  |  |
| Culture medium | TexMACS GMP | TexMACS GMP | Miltenyi Biotec |
| Interleuquin-2 | IL-2 | MACS GMP IL-2 | Miltenyi Biotec |
| anti-CD3/anti-CD28 Matrix | T cell TransAct | MACS GMP T cell TransAct | Miltenyi Biotec |
| Culture plastic | p24 well plate | 175 cm^2^ flask | Nunc/Thermo Scientific |

**Supplementary Table 1. Reagents and equipment equivalence between Research and GMP manufacturing protocol.**

| **Marker** | **Fluorochrome** | **Clone** |
| --- | --- | --- |
| **Surface** |  |  |
| CD3 | Viogreen | REA613 |
| CD4 | APC/Cy7 | 13B8.2 |
| CD8 | APC/Cy5 | B9.11 |
| CD25 | PC7 | M-A251 |
| CD27 | PC5.5 | 1A4CD27 |
| CD45RA | ECD | 2H4 |
| HLA-DR | PC5.5 | Immu357 |
| CD73 | BV605 | AD2 |
| LAG-3 | BV650 | 11C3C65 |
| CD39 | FITC | MZ18­23C8 |
| ICOS | BV650 | C398.4A |
| TIGIT | VioBright 515 | REA 1004 |
| GITR | PE-Vio615 | REA 1007 |
| LAP | APC | FNLAP |
| CCR4 | BV605 | L291H4 |
| CD62L | BV650 | DREG-56 |
| CXCR3 | PE-Vio615 | REA232 |
| **Intracellular** |  |  |
| CTLA4 | APC | BNI3 |
| FOXP3 | PE | PCH101 |
| HELIOS | PC5 | 22F6 |
| **Others** |  |  |
| Fixable Viability Dye | eFluor450 | - |

**Supplementary Table 2. Antibodies used for flow cytometric analysis.**
